# PREVALENCE AND INCIDENCE OF FOUR COMMON BEAN ROOT ROTS IN UGANDA

**DOI:** 10.1017/s0014479717000461

**Published:** 2017-09-25

**Authors:** P. PAPARU, A. ACUR, F. KATO, C. ACAM, J. NAKIBUULE, S. MUSOKE, S. NKALUBO, C. MUKANKUSI

**Affiliations:** † National Crops Resources Research Institute, Namulonge, PO Box 7084, Kampala, Uganda; § Centro Internacional de Agricultura Tropical (CIAT), PO Box 6247, Kampala, Uganda

## Abstract

Root rots are one of the main biotic constraints to common bean (*Phaseolus vulgaris* L.) production, causing losses estimated at 221 000 metric tons a year in sub-Saharan Africa. Until recently, root rots in Ugandan common bean agroecologies were mostly caused by *Pythium* and *Fusarium* spp., especially in high altitude areas. But now, severe root rots are observed in low and medium altitude agroecologies characterized by dry and warm conditions. The objective of our study was therefore to ascertain the current prevalence and incidence of common bean root rot diseases in Ugandan common bean agroecologies. Our results show that root rots were present in all seven agroecologies surveyed. Overall, the most rampant root rot was southern blight caused by *Sclerotium rolfsii* Sacc., followed by root rots caused by *Fusarium* spp., *Pythium* spp. and *Rhizoctonia solani*, respectively. Our study clearly showed the influence of environmental conditions on the prevalence and incidence of common bean root rots. While Fusarium and Pythium root rots are favoured under low air temperature and high air humidity in highland areas, high incidence of southern blight is favoured by warm and moist conditions of lowland areas. The prevalence and incidence of common bean root rots was mapped, providing a reliable baseline for future studies. Similarly, hotspots identified for common bean root rots will be a very useful resource for evaluation of germplasm and breeding lines for resistance to root rots.

## INTRODUCTION

The common bean (*Phaseolus vulgaris* L.) is an important food crop worldwide (Wortmann *et al.*, 1998), acclaimed for nutritive benefits such as high protein, micronutrients, vitamins and dietary fibre (Widders, 2006). The crop is the second most important source of calories after maize in sub-Saharan Africa, with over 200 million people depending on the crop as a primary staple (Akibode and Maredia, 2011). The production of common bean is; however, constrained by soil-borne pathogens that cause root rot. Common bean root rots cause significant yield losses and are widespread in Central and South America, and Africa (Abawi and Pastor Corrales, 1990; Buruchara *et al.*, 2015). The most common soil-borne pathogens that cause common bean root rots include *Pythium* spp., *Fusarium* spp., *Rhizoctonia solani* and *Sclerotium rolfsii* Sacc., the asexual stage of *Athelia rolfsii* (Curzi) Tu and Kimbrough. These four pathogens may cause losses of up to 100% in susceptible varieties under moist conditions and impoverished soils.

In Uganda, common bean root rots were previously known to be caused by *Pythium* and *Fusarium* spps., and the most severely affected areas were highlands, where air temperature is low and air relative humidity high (Buruchara and Rusuku, 1992; Opio *et al.*, 2007). Due to changing environmental conditions, such as higher temperatures and more frequent floods, the pattern of root rot diseases seems to be changing, with severe root rots occurring in low to mid-altitude areas. Farrow *et al.* (2011) predicted that the incidence of root rots would rise in East Africa due to higher rainfall during the cropping season. In the last 12 years, there has been no comprehensive research in Uganda to determine the incidence and severity of common bean root rot diseases and the contribution of the different root rot pathogens to the current disease upsurge. Past studies only focused on single pathogens (Mukalazi, 2004; Mukankusi, 2008; Tusiime, 2003). Similarly, earlier studies focused on very few common bean agroecologies. Wortmann and Eledu (1999) divided Uganda into 14 agroecological zones based on altitude, rainfall and soil type. However, Kalyebara *et al.* (2006) aggregated the 14 agroecologies into eight common bean agroecologies based on ecology, farming systems and the types of bean grown.

The objective of our study was to ascertain the current prevalence and incidence of common bean root rot diseases in seven of the eight common bean agroecologies in Uganda.

## MATERIALS AND METHODS

### Survey area

We conducted surveys in seven bean agroecologies over three growing seasons, March–June 2013 (2013A), August–November 2013 (2013B) and March–June 2014 (2014A). In 2013A, we surveyed 622 fields in seven bean agroecologies: Eastern Highlands (annual rainfall >1200 mm, altitude 1500–2200 m), Lake Victoria Basin and Mbale Farmlands (annual rainfall >1215–1328 mm, altitude 1040–1433 m), Northern Mixed Farming Zone (annual rainfall >1197 mm, altitude 942–1182 m), South Western Highlands (annual rainfall >1200 mm, altitude 1247–2313 m), Teso Farming System Zone (annual rainfall <1000mm, altitude 1072–1130 m), Western Mixed Farming System (annual rainfall >1000–1200 mm, altitude 1052–1501 m) and West Nile Mixed Farming System (annual rainfall >1340–1371 mm, altitude 918–1454 m). Temperature and rainfall data were obtained from www.erails.net/UG/aris/kms and presented in Supplementary Figures S1 and S2, while altitude is from GPS readings taken during our surveys. In 2013B, we surveyed 375 fields in the Northern Mixed Farming Zone, Western Mixed Farming System, Lake Victoria Basin and Mbale Farmlands and South Western Highlands. In 2014A, we surveyed 120 fields in the Western Mixed Farming System, Lake Victoria Basin and Mbale Farmlands, and South Western Highlands. Thus, the total number of fields surveyed was 1117. Though some agroecologies were surveyed more than once, different fields were surveyed during each visit.

In each agroecology, we chose districts to survey based on common bean production records published by Kalyebara *et al.* (2006). Districts with high common bean production rates and those where the crop in the field was at a suitable stage for root rot disease assessment were surveyed. In each district, we surveyed two subcounties. In each sub-county, 20 fields randomly chosen at distances of 1–5 km along the survey route were surveyed. A survey route here refers to gravel roads in the chosen villages that could be accessed by our field vehicle. However, in some districts, we surveyed fewer fields because the crop was not at a suitable stage for assessing root rot disease. The average size of farms surveyed in all agroecologies was 0.7 ha. In the fields sampled, the growth stage of the crop ranged from primary leaf stage to pod initiation. Field size was measured using GPS and the prevalence and incidence of root rot was assessed through field observation.

### Data collected

To determine the prevalence of root rot disease, we walked along a Z transect in each field and observed the presence or absence of wilting due to common bean root rot diseases. Disease prevalence refers to the incidence of diseased plants in a defined geographic area (Nutter *et al.*, 1991). To determine root rot disease incidence, we randomly selected a minimum of 20 wilting plants showing the characteristic symptoms of root rot diseases along the Z transect. Disease incidence refers to the number of sampled plants that are diseased, expressed as percentage of the total number of samples (Nutter *et al.*, 1991). We then identified the symptoms shown by each wilting plant using the guidelines in the Centro Internacional de Agricultura Tropical (CIAT) handbook for bean disease and pest identification (Buruchara *et al.*, 2010). The symptoms we used to identify the pathogens were: (1) for *Fusarium* spp. root rot – narrow, longitudinal reddish-brown lesions on hypocotyl, accompanied by longitudinal fissures or cracks; (2) for *Pythium* spp. root rot – elongated water-soaked areas on hypocotyl appearing 1–3 weeks after planting, dry and brown lesions; (3) for *Sclerotium rolfsii* root rot (southern blight) – grey water-soaked lesions on hypocotyl just below the soil line, later becoming brown and extending to the tap root, abundant silky mycelium and large round sclerotia at the base of the stem and (4) for *Rhizoctonia solani* root rot – dark, circular and oblong cankers delimited by brown margins on the hypocotyl, later becoming red, rough, dry and pithy. Diseased plant samples were collected for each root rot disease and used for pathogen isolation in the laboratory (data not shown).

### Data analysis

We computed the mean percentage prevalence and incidence of common bean root rots by season, agroecology and district, and performed analysis of variance (ANOVA) for percentage prevalence and incidence of common bean root rots using the general linear model (GLM). Statistical differences between districts were determined using Fisher’s Protected Least Significant difference test. Differences for seasons and agroecologies were separated using Tukey Studentized range test. For all tests, significance was evaluated at *p* ≤ 0.05. All analyses were performed using Statistical Analysis Systems version 9.1 (SAS Institute, Cary NC, USA).

## RESULTS

### Root rot prevalence

**Figure 1.**
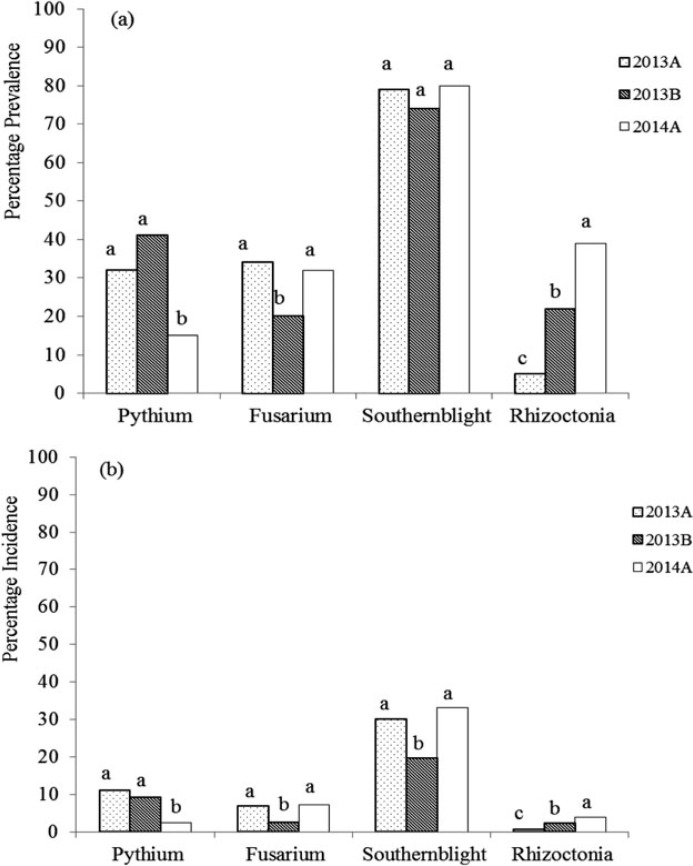
Seasonal prevalence (a) and incidence (b) of Pythium, Fusarium, Sclerotium and Rhizoctonia root rots in Ugandan common bean agroecologies across three seasons (2013A, 2013B and 2014A) between March 2013 and June 2014. For each root rot type, bars followed by different letters are significantly different (*p* ≤ 0.05, Tukey’s Studentized range test).

Common bean root rots were observed in all sampled agroecologies (Table 1). However, the prevalence of root rots varied across seasons (Figure 1a). For agroecologies sampled in two or three seasons, the prevalence of Pythium root rot was different across seasons (F = 14.72, *p* < 0.0001) (Figure 1a). The same was true for Fusarium (F = 11.33, *p* < 0.0001) and Rhizoctonia (F = 59.88, *p* < 0.0001) root rots (Figure 1a). On the other hand, the prevalence of southern blight did not vary across seasons (F = 2.10, *p* = 0.12). Although more than half the fields surveyed (52%) were affected by a single root rot pathogen, several fields were affected by more than one root rot pathogen. The percentage of fields affected by two, three or four root rot pathogens simultaneously was 34, 10 and 1%, respectively. Individual plants were also infected by more than one root rot pathogen.

**Table 1.** Percentage prevalence and incidence of common bean root rots in seven agroecologies surveyed across three seasons (2013A, 2013B and 2014A) between March 2013 and June 2014.

Percentage prevalence of root rots†,‡					Percentage incidence of root rots†,‡				
Agroecology^*^	season	Pythium	Fusarium	Southern blight	Rhizoctonia	Pythium	Fusarium	Southern blight	Rhizoctonia
EH	2013A	0.0 c	50.0 ab	83.3 a	0.0 a	0.0 b	7.5 abc	17.1 bc	0.0 a
LVC	2013A	14.9 bc^AB^	14.5 dc^A^	89.6 a^AB^	3.3 a ^B^	1.0 b^A^	2.0 bc^A^	34.2 ab^B^	0.7 a^B^
NM	2013A	17.8 bc^A^	22.2 bcd^A^	88.9 a^A^	4.4 a^A^	1.8 b^B^	4.1 bc^A^	43.9 a^A^	0.2 a^A^
SWH	2013A	75.7 a^A^	65.6 a^A^	46.1 b^A^	7.8 a^C^	35.3 a^A^	13.9 a^A^	6.9 c^B^	0.9 a^B^
TFS	2013A	87.5 a	6.3 d	75 a	0.0a	4.4b	0.3 c	3.8 c	0.0 a
WM	2013A	27.1 b^B^	47.9 ab^A^	90.6 a^A^	5.2 a^B^	6.1 b^AB^	10.8 ab^A^	38.2 a^A^	0.5 a^B^
WNM	2013A	4.2 bc	38.9 abc	90.3 a	0.0a	0.3b	6.8 abc	41.1 a	0.0 a
LVC	2013B	24.6 b^A^	14.0 b^A^	82.5 ab^B^	28.1 a^A^	2.3 c^A^	1.9 b^A^	20.7 b^C^	3.1 a^A^
NM	2013B	25.5 b ^A^	8.5 b^B^	86.6 a^A^	13.4 a^A^	10.0 b^A^	1.1 b^B^	34.9 a^A^	1.5 a^A^
SWH	2013B	80.3 a^A^	47.0 a^A^	39.4 c^A^	22.7 a^B^	23.8 a^B^	6.8 a^B^	4.4 c^B^	2.8 a^B^
WM	2013B	67.3 a^A^	23.6 b^A^	67.3 b^B^	16.4 a^B^	12.1 b^A^	1.9 b^A^	11.5 c^B^	1.1 a^B^
LVC	2014A	3.8 b^B^	12.5 b^A^	98.1 a^A^	26.7 a ^A^	1.1 a^A^	2.2 b^A^	50.0 a^A^	2.1 a^AB^
SWH	2014A	33.3 a^B^	58.3 a^A^	54.2 b^A^	54.2 a^A^	4.9 a^C^	4.3 a^B^	18.0 b^A^	6.1 aA
WM	2014A	0.0 b^C^	23.5 b^A^	94.1 a^A^	35.3 a^A^	0.0 a^B^	3.5 b^A^	19.7 b^B^	3.1 a^A^

* EH = Eastern Highlands, LVC = Lake Victoria Basin and Mbale Farmlands, NM = Northern Mixed Farming Zone, SWH = South Western Highlands, TFS =Teso Farming System Zone, WM = Western Mixed Farming System and WNM = West Nile Mixed Farming System.

† For each season, means within a column followed by different lower case letters are significantly different (*p* ≤ 0.05, Tukey’s Studentized range test).

‡ For each agroecology surveyed more than once means within a column followed by different upper case superscript letters are significantly different across seasons (*p* ≤ 0.05, Tukey’s Studentized range test).

Analysis by season showed significant differences in the prevalence of common bean root rots in Ugandan bean agroecologies. Whereas the prevalence of Pythium, Fusarium and Sclerotium root rots was significantly different across agroecologies surveyed in 2013A (F = 47.41, *p* < 0.001; F = 23.53, *p* < 0.001; F = 24.80, *p* < 0.001 for Pythium, Fusarium and Sclerotium root rots, respectively), the prevalence of Rhizoctonia root rot was not (F = 1.58, *p* = 0.15). The highest prevalence of Pythium root rot was in the Teso Farming Systems Zone (87.5%), followed by the South Western Highlands (Table 1). The highest prevalence of Fusarium root rot was in the South Western Highlands, followed by the Eastern Highlands and these were significantly higher than prevalence in three other agroecologies surveyed in the same season (Table 1). Apart from the South Western Highland agroecology that showed the least prevalence of southern blight (46.1%), prevalence in the other six agroecologies was between 75–90.6% in 2013A (Table 1).

As in 2013A, the prevalence of Pythium, Fusarium and Sclerotium root rots was significantly different across agroecologies in 2013B (F = 36.29, *p* < 0.001; F = 15.15, *p* < 0.001; F = 21.17, *p* < 0.001 for Pythium, Fusarium and Sclerotium root rots, respectively). Again the prevalence of Rhizoctonia root rot was not significantly different across agroecologies (F = 2.71, *p* = 0.054). The highest prevalence of Pythium root rot was in the South Western Highlands and in the Western Mixed Farming System (Table 1). Fusarium root rot was most prevalent in the South Western Highlands, and this was significantly higher than prevalence in other agroecologies (Table 1). The highest prevalence of southern blight was observed in the Northern Mixed Farming Zone, whereas the lowest prevalence occurred in the South Western Highlands.

In 2014A, the highest prevalence of both Pythium and Fusarium root rots occurred in the South Western Highlands (Table 1). As found in 2013A and 2013B, the South Western Highlands registered the lowest prevalence of southern blight in 2014A. However, the highest prevalence of southern blight was observed in the Lake Victoria Basin and Mbale Farmlands agroecology.

Significant differences were observed in the prevalence of common bean root rots among the districts in different agroecologies (Supplementary Table S3). In districts such as Bugiri, Busia, Butalejja and Jinja in the Lake Victoria Basin and Mbale Farmlands agroecology, the prevalence of southern blight was 100% (all farms surveyed had beans wilting as a result of southern blight). While southern blight was present in all districts surveyed, the other three root rots were not observed in some districts. For example, Pythium root rot was absent in the neighbouring districts of Bugiri, Busia, Butalejja, Jinja and Namutumba and in Lira and Oyam. Similarly, Fusarium root rot was absent in the districts of Nakaseke, Mukono and Namutumba. Rhizoctonia root rot was also not observed in all the districts surveyed in West Nile Mixed Farming System agroecology.

### Root rot disease incidence

The incidence of bean root rots also varied across seasons (Figure 1b). For agroecologies sampled in two or three seasons, incidence of Pythium root rot was different across seasons (F = 8.02, *p* = 0.0004). The same was true for Fusarium (F = 14.04, *p* < 0.0001), Sclerotium (F = 15.09, *p* < 0.0001) and Rhizoctonia (F = 20.05, *p* < 0.0001) root rots.

**Figure 2.**
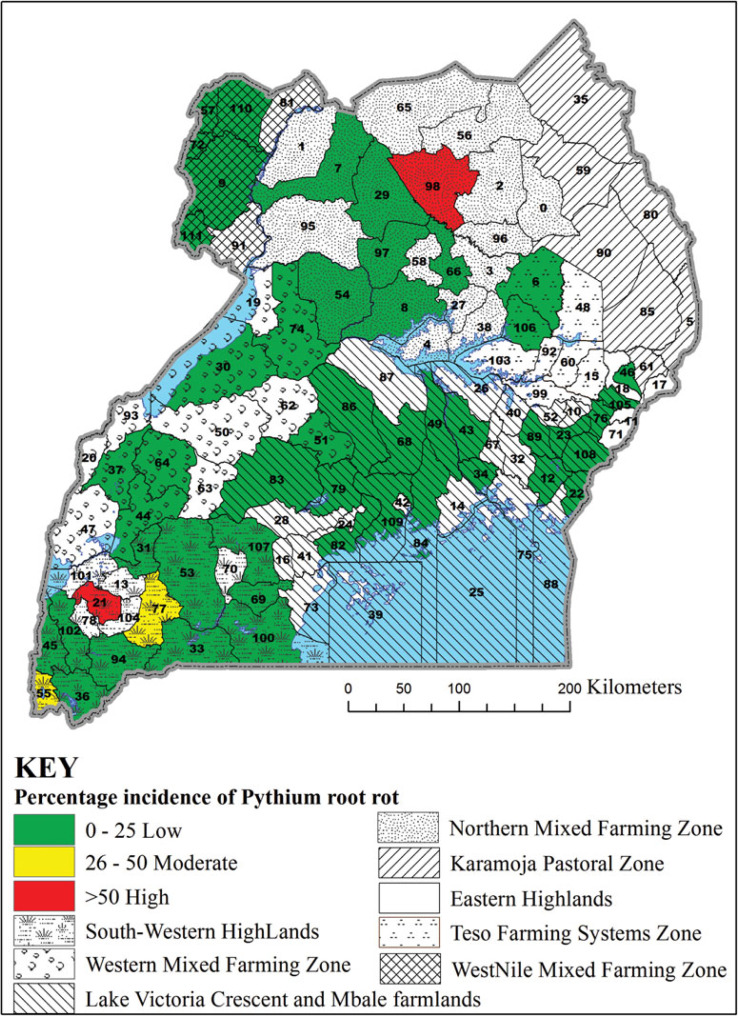
Incidence of Pythium root rot in selected districts in seven common bean agroecologies in Uganda. District 6 = Amuria, 7 = Amuru, 8 = Apac, 9 = Arua, 12 = Bugiri, 21 = Bushenyi, 22 = Busia, 23 = Butaleja, 30 = Hoima, 31 = Ibanda, 33 = Isingiro, 34 = Jinja, 36 = Kabale, 43 = Kamuli, 44 = Kamwenge, 46 = Kapchorwa, 49 = Kayunga, 51 = Kiboga, 53 = Kiruhura, 54 = Kiryadongo, 55 = Kisoro, 56 = Kitgum, 57 = Koboko, 64 = Kyenjojo, 66 = Lira, 68 = Luwero, 69 = Lwengo, 72 = Maracha, 74 = Masindi, 76 = Mbale, 77 = Mbarara, 79 = Mityana, 82 = Mpigi, 83 = Mubende, 84 = Mukono, 86 = Nakaseke, 94 = Ntungamo, 97 = Oyam, 98 = Pader, 100 = Rakai, 102 = Rukungiri, 105 = Sironko, 109 = Wakiso, 110 = Yumbe and 111 = Zombo.

**Figure 3.**
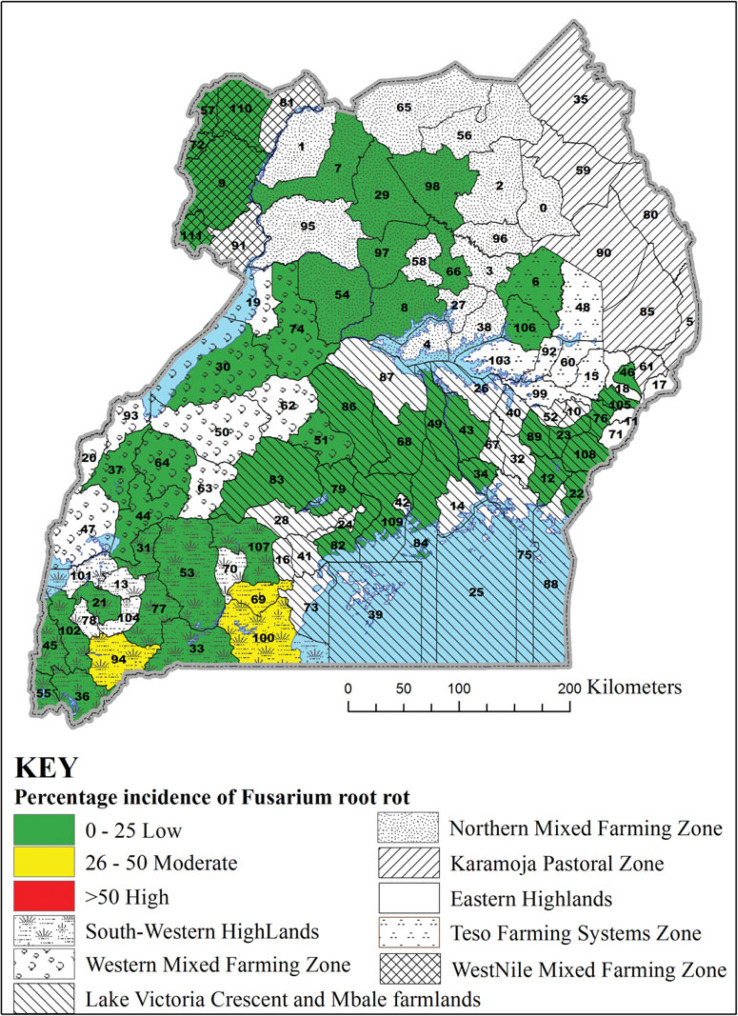
Incidence of Fusarium root rot in selected districts in seven common bean agroecologies in Uganda. District 6 = Amuria, 7 = Amuru, 8 = Apac, 9 = Arua, 12 = Bugiri, 21 = Bushenyi, 22 = Busia, 23 = Butaleja, 30 = Hoima, 31 = Ibanda, 33 = Isingiro, 34 = Jinja, 36 = Kabale, 43 = Kamuli, 44 = Kamwenge, 46 = Kapchorwa, 49 = Kayunga, 51 = Kiboga, 53 = Kiruhura, 54 = Kiryadongo, 55 = Kisoro, 56 = Kitgum, 57 = Koboko, 64 = Kyenjojo, 66 = Lira, 68 = Luwero, 69 = Lwengo, 72 = Maracha, 74 = Masindi, 76 = Mbale, 77 = Mbarara, 79 = Mityana, 82 = Mpigi, 83 = Mubende, 84 = Mukono, 86 = Nakaseke, 94 = Ntungamo, 97 = Oyam, 98 = Pader, 100 = Rakai, 102 = Rukungiri, 105 = Sironko, 109 = Wakiso, 110 = Yumbe and 111 = Zombo.

**Figure 4.**
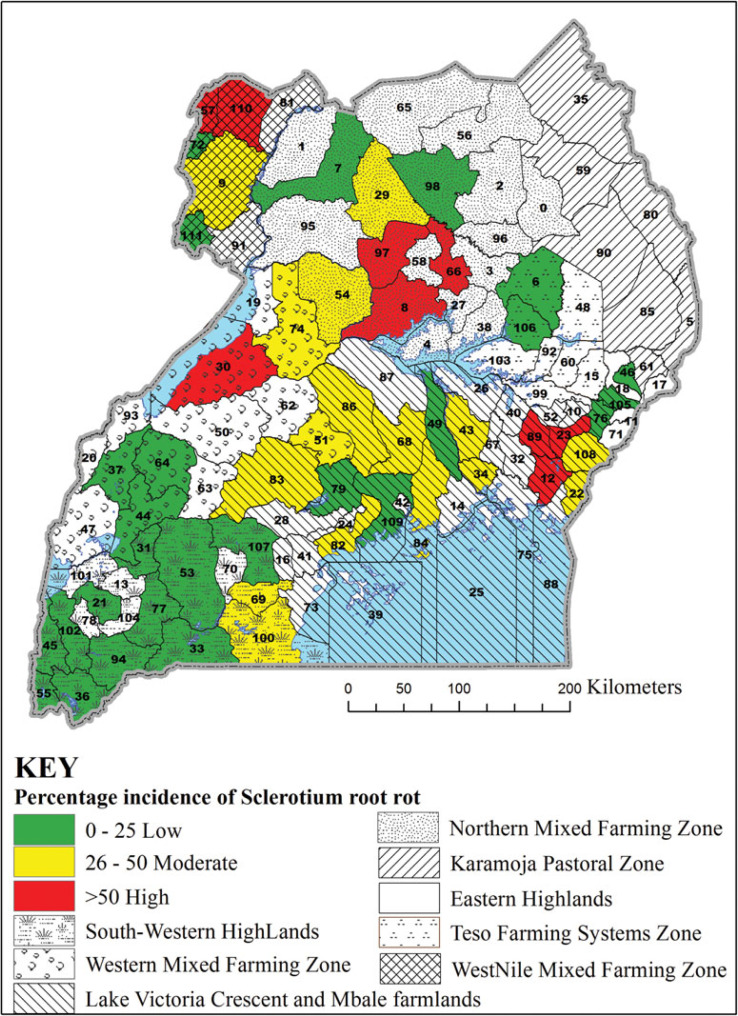
Incidence of southern blight in selected districts in seven common bean agroecologies in Uganda. District 6 = Amuria, 7 = Amuru, 8 = Apac, 9 = Arua, 12 = Bugiri, 21 = Bushenyi, 22 = Busia, 23 = Butaleja, 30 = Hoima, 31 = Ibanda, 33 = Isingiro, 34 = Jinja, 36 = Kabale, 43 = Kamuli, 44 = Kamwenge, 46 = Kapchorwa, 49 = Kayunga, 51 = Kiboga, 53 = Kiruhura, 54 = Kiryadongo, 55 = Kisoro, 56 = Kitgum, 57 = Koboko, 64 = Kyenjojo, 66 = Lira, 68 = Luwero, 69 = Lwengo, 72 = Maracha, 74 = Masindi, 76 = Mbale, 77 = Mbarara, 79 = Mityana, 82 = Mpigi, 83 = Mubende, 84 = Mukono, 86 = Nakaseke, 94 = Ntungamo, 97 = Oyam, 98 = Pader, 100 = Rakai, 102 = Rukungiri, 105 = Sironko, 109 = Wakiso, 110 = Yumbe and 111 = Zombo.

In 2013A, the incidence of Pythium root rot was different among agroecologies surveyed (F = 64.61, *p* < 0.0001), being highest in the South Western Highlands (Table 1). Unexpectedly, the highest incidence of Pythium root rot (71%) among the districts surveyed was in Pader in the Northern Mixed Farming Zone, which is in a warm and low altitude zone (Figure 2). The second highest incidence of Pythium root rot disease (53%) was in Bushenyi district in the cool and high altitude South Western Highlands agroecology. Similarly, significant differences were observed in the incidence of Fusarium root rot among agroecologies in 2013A (F = 13.65, *p* < 0.0001) (Table 1), with the highest incidence registered in the South Western Highlands agroecology. However, two neighbouring districts of Rakai and Lwengo in the Lake Victoria Basin and Mbale Farmlands agroecology presented the highest (about 38%) incidences of Fusarium root rot (Figure 3). In Kanungu in the South Western Highlands, incidence of Fusarium root rot was 23%. The incidence of southern blight also varied across agroecologies surveyed in 2013A (F = 21.41, *p* < 0.0001), with the highest incidence observed in the Northern Mixed Farming Zone, the West Nile Mixed Farming System and Western Mixed Farming System (Table 1). Bugiri district in Lake Victoria Basin and Mbale Farmlands registered the highest incidence of southern blight (84%) and other districts with high southern blight incidences were Koboko, Hoima, Oyam and Lira (Figure 4).

The incidence of Pythium, Fusarium and Sclerotium root rots was also different across agroecologies surveyed in 2013B (F = 29.33, *p* < 0.001; F = 9.92, *p* < 0.001; F = 27.49, *p* < 0.001 for Pythium, Fusarium and Sclerotium root rots, respectively). Again, the incidence of Rhizoctonia root rot did not vary across agroecologies (F = 2.54, *p* = 0.056). The highest incidence of both Pythium and Fusarium root rots was observed in the South Western Highlands and this was significantly higher than that for three other agroecologies surveyed in 2013B (Table 1). The highest incidence of southern blight was noticed in the Northern Mixed Farming Zone (Table 1). In 2014A, the highest incidence of both Pythium and Fusarium root rots still occurred in the South Western Highlands (Table 1). However, the highest incidence of Southern blight was in the Lake Victoria Basin and Mbale Farmlands agroecology.

## DISCUSSION

Our study is the first known systematic survey to determine the prevalence and incidence of common bean root rots in Uganda. The survey found that root rots were present in all seven common bean agroecologies and the occurrence of more than one root rot disease on a single plant was observed. Where symptoms were vague or in an initial stage for the second or third pathogen on a single plant, an underestimation of prevalence and incidence may have occurred. However, symptoms of root rots observed in the field were characteristic of the pathogens *Fusarium* spp., *Pythium* spp., *Rhizoctonia solani* and *Sclerotium rolfsii* and we did not look for root rot diseases other than the above. We confirmed our field observations by isolating the pathogens from wilting plants (data not shown). Notably, wilting in several instances was caused by the bean stem maggot (*Ophiomyia phaseoli*).

The prevalence of the different bean root rots varied among bean agroecologies. A high prevalence of Pythium root rot was observed in the Teso Farming Systems Zone and South Western Highlands (Table 1). However, the high prevalence of Pythium root rot in Teso Farming Systems Zone needs to be confirmed because we assessed root rot disease in only 18 fields. In addition, the surveys in the Teso Farming Systems Zone were conducted only in a single season and a second data collection is needed in order to draw meaningful conclusions. Fusarium root rot was most common in the South Western Highlands and the Eastern Highlands (Table 1), confirming earlier findings by Tusiime (2003). The most common root rot was southern blight with 77–90% prevalence in most agroecologies (Table 1). The highest prevalence of southern blight was in the West Nile Mixed Farming System and the lowest in the South Western Highlands (Table 1).

Interestingly, the incidence of root rots followed the same pattern as their prevalence (Table 1). The high prevalence and incidence of Fusarium and Pythium root rots in the South Western Highlands may be because *Fusarium* and *Pythium* spp. pathogens thrive in similar environments. According to Abawi and Pastor Corrales (1990), a synergistic interaction between *Fusarium solani* f.sp. *phaseoli* and *Pythium ultimum* results in greater damage than the action of each pathogen alone. Environmental conditions, soil and cropping systems may also influence the severity of common bean root rots across regions (Abawi and Pastor Corrales, 1990). The same phenomenon has been reported for other crops and types of wilt. For example, the incidence of Phytophthora blight and Verticillium wilt in pepper varied across regions and was believed to be influenced by environmental conditions and cropping practices (Sanoga and Carpenter, 2006). Our study clearly showed the influence of environmental conditions on the prevalence and incidence of the different bean root rots: Fusarium and Pythium root rots are favoured by cool temperatures and high air humidity of highland areas and southern blight by the warm and moist conditions in lowland areas.

The identification of southern blight as the most common root rot is significant because *Sclerotium rolfsii* also causes root and stem rots in crops such as groundnut (Le *et al.*, 2012), maize (Ahmed *et al.*, 1984), tomato, onion and soybean (Flores-Moctezuma *et al.*, 2006), which are commonly grown in Ugandan common bean production regions. Our study showed that farmers commonly rotate beans with crops such as groundnuts and maize, hence perpetuating the occurrence of southern blight in their fields. Other crops have also been shown to harbour pathogens that cause bean root rot and possibly act as alternate hosts (Gichuru, 2008). Our study has identified hot spots for bean root rot diseases (Figures 2, 3 and 4). These are locations where high prevalence and incidence of the different root rots was observed. For example, districts such as Bugiri, Hoima, Koboko and Oyam could be marked as hot spots for southern blight (Figure 4), while Lwengo, Ntungamo and Rakai for Fusarium root rot (Figure 3), and Bushenyi, Kisoro and Mbarara for Pythium root rot (Figure 2). Pathologists and plant breeders can use such hot spots for evaluation of germplasm and breeding lines.

This study provides a baseline for mapping changes in the prevalence and incidence of common bean root rots in future. Due to its importance in forecasting and setting disease management priorities, regular disease mapping is becoming increasingly vital because of global environmental changes that greatly impact the occurrence of crop diseases.

## CONCLUSIONS

The results of the surveys we conducted show that root rots are still a major constraint to common bean production in Uganda. Whereas the prevalence and incidence of previously studied root rots such as Pythium and Fusarium have not changed significantly, there is a high prevalence and incidence of southern blight in several common bean producing districts. The latter therefore necessitates further investigations to determine the effect of the observed root rot occurrences on crop losses, and to develop or validate root rot management practices that can reduce losses on-farm as well as developing and releasing resistant varieties.

## SUPPLEMENTARY MATERIAL

To view supplementary material for this article, please visit https://doi.org/10.1017/S0014479717000461
